# Taurine Alleviates Inflammation, Oxidative Stress, Apoptosis, and Uterus Microbiota Dysregulation of Endometritis by Inhibiting PI3K-AKT/MAPK/NF-κB Pathways in Mice

**DOI:** 10.3390/ani15243619

**Published:** 2025-12-16

**Authors:** Jianxu Xiao, Chongliang Bi, Ming Yang, Chen Chen, Juanjuan Zhao, Xiaoqing Huang, Jingyuan Zhang, Buwei Yin, Ke Li, Yuzhong Ma

**Affiliations:** 1 College of Veterinary Medicine, Hebei Agricultural University, Baoding 071000, China; xiaojianxu1819@126.com (J.X.); 19943780686@163.com (C.C.); 18614033676@163.com (J.Z.); m18232799556@163.com (X.H.); zzzjy521@163.com (J.Z.); yinbuwei@gmail.com (B.Y.); 2 College of Agriculture and Forestry Science, Linyi University, Linyi 276005, China; bichongliang@lyu.edu.cn; 3 College of Veterinary Medicine, Shandong Agricultural University, Taian 271018, China; 15076077823@163.com

**Keywords:** bovine endometritis, taurine, LPS, uterine microbiota, PI3K-AKT/MAPK/NF-κB pathway

## Abstract

Bovine endometritis negatively impairs fertility and milk production. Taurine maintains cellular integrity and exerts anti-inflammatory and antioxidant effects. However, whether taurine can treat endometritis remains unclear. This study aimed to investigate taurine’s effect on endometritis and explore its mechanism in vivo. Endometritis models were established in mice via intrauterine lipopolysaccharide (LPS) infusion, followed by 25, 50, and 100 mg/kg taurine treatment. Taurine attenuated inflammation by mitigating histopathological damage, suppressing uterine serum cytokine levels, and preserving tight-junction integrity. It ameliorated oxidative stress by reducing malondialdehyde content, restoring antioxidant activities, and recovering levels of oxidative-stress-related proteins. Apoptosis was alleviated by diminishing the apoptosis ratio and normalizing apoptosis-related proteins. 16S analysis revealed taurine restored uterine microbiota composition by reversing the changes in the abundances of Firmicutes, Bacteroidetes, Nocardioides, Ruminococcus, and Acidibacter. The abundances of Muribacter and Rodentibacter were positively correlated with inflammation. The abundances of Akkermansia and Streptococcus were negatively correlated with inflammation. RNA sequencing showed that the differentially expressed genes were mainly related to immunity. Phosphatidylinositol 3-kinase (PI3K)-protein kinase B (AKT)/mitogen-activated protein kinase (MAPK)/nuclear factor kappa B (NF-κB) signaling pathways were indicated as pivotal mechanisms for taurine’s therapeutic efficacy against endometritis with transcriptomic profiling analysis. This study confirms that taurine alleviates LPS-induced endometritis in mice by modulating PI3K–AKT, MAPK, and NF-κB signaling pathways, indicating its potential as a therapeutic agent for bovine endometritis.

## Simple Summary

Bovine endometritis impairs the reproductive function of dairy cows and reduces milk production, causing substantial economic losses. This study aimed to investigate the preventive effect of taurine on bovine endometritis, using a lipopolysaccharide (LPS)-induced mouse model of endometritis for experiments. The results showed that taurine could effectively alleviate endometritis symptoms in model mice: it not only reduced oxidative stress damage, inhibited inflammation, and decreased apoptosis, but also regulated the structure of the uterine microbiota to restore microbial balance. Further mechanistic studies confirmed that taurine alleviated LPS-induced endometritis by regulating Phosphatidylinositol 3-kinase (PI3K)-protein kinase B (AKT)/mitogen-activated protein kinase (MAPK)/nuclear factor kappa B (NF-κB) signaling pathways. In conclusion, taurine is expected to become a potential therapeutic agent for bovine endometritis. This finding holds an important reference value for improving the diagnosis and treatment of inflammatory diseases in the veterinary field.

## 1. Introduction

Bovine endometritis, as one of the most common postpartum reproductive disorders in multiparous dairy cows, significantly impairs fertility and milk production [1]. Postpartum endometrial injury in dairy cows is usually induced by placental detachment, which creates optimal conditions for pathogen infiltration into the uterus, particularly by Gram-negative organisms such as *Escherichia coli* [2]. Lipopolysaccharides (LPS), a component of *Escherichia coli*, is specifically recognized and bound by Toll-like receptors of the innate immune system to enable intracellular signal transduction. Both nuclear factor kappa B (NF-κB) and mitogen-activated protein kinase (MAPK) signaling pathways are activated through intricate cascade reactions, and pro-inflammatory cytokines and chemokines are secreted, leading to inflammatory damage in uterine tissues [3]. Subsequently, accumulation of reactive oxygen species (ROS) is induced by the release of pro-inflammatory mediators, leading to depletion of antioxidants, including glutathione (GSH) and superoxide dismutase (SOD) [4]. Additionally, mitochondrial dysfunction is induced by ROS, characterized by dysregulation of apoptosis-related proteins, which activates the cysteine-aspartic acid protease-3 (Caspase-3) cascade, initiating apoptosis [5]. It exacerbated uterine dysfunction, manifesting as impaired contractility and decreased mucin production [6].

Endometritis is particularly prevalent and imposes substantial economic losses on the dairy industry due to high incidence rates in multiparous cows. Current therapeutic drugs for endometritis predominantly rely on antibiotics; however, prolonged antibiotic use risks bacterial resistance and drug residues in milk [7]. Therefore, it is necessary to explore novel treatment approaches for endometritis. Studies indicate that amino acids maintain immune homeostasis and exhibit anti-inflammatory capacity by modulating immunoglobulin synthesis, supporting immune cell function, influencing immune cell differentiation, and regulating inflammatory cytokine expression [8,9]. Taurine (β-aminoethanesulfonic acid), a critical endogenous antioxidant, scavenges free radicals and lipid peroxides while stabilizing cell membranes, thereby maintaining cellular integrity and exerting antioxidant effects [10]. Additionally, taurine inhibits inflammatory mediator production and modulates the NF-κB signaling pathway, ultimately attenuating inflammation [11]. The potential therapeutic and prophylactic value of taurine for disease is highlighted by these properties. Nevertheless, research on taurine for treating bovine endometritis remains limited.

Therefore, this study aimed to investigate the protective effects of taurine against LPS-induced endometritis in mice and explore its mechanism, thus providing a theoretical foundation for the prevention and treatment of bovine endometritis.

## 2. Materials and Methods

### 2.1. Chemicals

Taurine (C_2_H_7_NO_3_S, MW 125.15, purity ≥ 98%) and LPS (*E. coli* O111:B4, purity ≥ 98%) were purchased from Sigma–Aldrich (Sigma–Aldrich, St. Louis, MO, USA).

### 2.2. Animals and Experiment Design

Forty specific-pathogen-free-grade female Kunming mice (7–8 weeks old, 30–35 g) were purchased from Liaoning Changsheng Biotechnology Corporation (Benxi, China). Water and food were provided ad libitum. Animal assays were approved by the Laboratory Animal Ethics Committee of Hebei Agricultural University (protocol number 2025066). Mice were randomly divided into five experimental groups (*n* = 8): (1) control group, (2) LPS group, (3) LPS + 25 mg/kg taurine (Tau) group, (4) LPS + 50 mg/kg taurine group, and (5) LPS + 100 mg/kg taurine group. The control group received saline treatment. For the other groups, 50 μL of LPS solution (1 g/L) was slowly injected into each uterine horn. Taurine (25, 50, or 100 mg/kg) was administered via intraperitoneal injection 1 h before LPS infusion. Twenty-four hours after LPS administration, all mice were euthanized by cervical dislocation, and uterine tissues were collected.

### 2.3. Pathological Lesions and Uterine Tissue Evaluation

After euthanasia, the abdominal skin of mice was incised along the midline, and the liver, spleen, and uterine tissues were excised and weighed. Pathological changes in the uterus were evaluated according to the report of Li et al. [12] using a clinical scoring system (1–5): 1 = no injury, 2 = mild redness and swelling, 3 = mild redness and swelling with minor hemorrhage, 4 = moderate redness and swelling with hemorrhage, and 5 = severe redness and swelling with hemorrhage. Uterine lesions were independently scored, with higher scores representing more severe tissue damage.

### 2.4. Uterine Tissue Section Preparation and Histopathological Analysis

Uterine tissues were fixed in 4% paraformaldehyde for 24 h, then trimmed and rinsed under running water for 24 h. Subsequently, tissues were dehydrated in a graded ethanol series (70%, 80%, 90%, and 100%), cleared in xylene, embedded in paraffin and sectioned at 4–5 μm thickness. After deparaffinization, sections were stained with hematoxylin and eosin (HE) and mounted with neutral resin. Histopathological changes were evaluated under a light microscope (Olympus, Tokyo, Japan). Histopathological scores were evaluated according to the methods reported by Guo et al. and Su et al. [13,14]. The scores consisted of 5 grades with the following criteria: 1. No inflammation. 2. Low infiltration of white blood cells and lymphocytes, with no structural changes. 3. Moderate infiltration of white blood cells and lymphocytes, capillary congestion. 4. Heavy infiltration of white blood cells and lymphocytes, capillary congestion, severe deformation of uterine glands, accompanied by substantial deposition of eosinophilic secretions. 5. Extensive infiltration of white blood cells, high vascular density, severe collapse of uterine glands, accompanied by abundant deposition of eosinophilic secretions, and disappearance of endometrial epithelial structure.

### 2.5. Myeloperoxidase Activity Assay

Uterine tissues were homogenized in normal saline at a weight-to-volume ratio of 1:19 to prepare 5% tissue homogenates. Myeloperoxidase (MPO) activities were measured using an MPO assay kit (Jiancheng Technology, Nanjing, China) according to the manufacturer’s instructions. Absorbance at 460 nm was measured using a microplate reader (Biotek, Winooski, VT, USA).

### 2.6. ELISA Assay

Blood samples were collected from the mice and centrifuged to analyze serum inflammatory cytokine levels. Levels of tumor necrosis factor alpha (TNF-α), interleukin-1β (IL-1β), and interleukin-6 (IL-6) were quantified using commercial assay kits (LunChangShuo Biotech, Xiamen, China) according to the manufacturer’s instructions. Absorbances were measured at 450 nm using a microplate reader (Biotek, Winooski, VT, USA).

### 2.7. Antioxidant-Related Enzyme Activities Measurement

Uterine tissues were homogenized in saline (0.9% NaCl) at a weight-to-volume ratio of 1:9 to prepare 10% tissue homogenates, and the supernatants were collected by centrifugation of the homogenates. Levels of MDA, GSH, T-AOC, and SOD were quantified using commercial assay kits (Jiancheng Technology, Nanjing, China) according to the manufacturer’s instructions. Absorbances were measured at 532, 405, 593, and 450 nm using a microplate reader (Biotek, Winooski, VT, USA).

### 2.8. TUNEL Assay

Paraffin-embedded tissue sections were sequentially subjected to xylene dewaxing followed by graded ethanol hydration. Apoptosis of uterine tissues was measured using a TUNEL cell apoptosis kit (Yeasen Biotechnology, Shanghai, China) according to the manufacturer’s instructions. Fluor microscopy (Zeiss, Oberkochen, Germany) was used to analyze samples with filter sets for green fluorescence at 520 nm and blue DAPI fluorescence at 460 nm.

### 2.9. Reverse Transcription Real-Time Quantitative PCR Analysis

Uterine tissues were minced and homogenized in TransZOL Up reagent (TransGen Biotech, Beijing, China), and total RNA was extracted. RNA concentration and purity were determined using a Nanodrop 2000 instrument (Thermo Fisher Scientific, Waltham, MA, USA). Subsequently, two micrograms of RNA were reverse transcribed into cDNA using a TransScript cDNA Synthesis Kit (TransGen Biotech, Beijing, China). Real-time PCR was performed using a fluorescence quantitative PCR system (Applied Biosystems, Carlsbad, CA, USA), and cycle threshold values were obtained. Relative expression levels were calculated using the 2^−∆∆Ct^ method. Primers were synthesized by Sangon Biotech (Shanghai, China), and sequences were provided in Table 1.

### 2.10. 16S rRNA Sequencing Analysis

Genomic DNA was extracted from uterine tissues and subjected to sequencing on the Illumina PE 250 platform (Novogene, Beijing, China). Following amplification and quality verification, PCR products were pooled and purified. The library preparation process included end repair, tailing, adapter ligation, and additional purification steps. Raw sequencing data were processed using FLASH (v1.2.11) to merge paired-end reads (R1 and R2), generating merged Tags. Subsequently, denoising was performed using DADA2 to derive amplicon sequence variants (ASVs), which served as high-resolution analogs to traditional operational taxonomic unit (OTU) representative sequences. Finally, taxonomic annotation and abundance analysis were conducted on the processed ASV data.

### 2.11. RNA-Sequencing Analysis

Total RNA was isolated from tissue samples, and RNA integrity was assessed using an Agilent 2100 Bioanalyzer (Agilent Technologies, Walnutt, CA, USA). Sequencing libraries were prepared following the NEB standard protocol, which included mRNA enrichment, reverse transcription, cDNA synthesis, end repair, and PCR amplification. The insert size of each library was verified before pooling at equimolar ratios based on effective concentration and target sequencing depth for Illumina sequencing. Gene expression levels were quantified using the featureCounts tool from the subread package, and differentially expressed genes (DEGs) were identified using stringent criteria (|log2(FoldChange)| ≥ 1 and padj ≤ 0.05) for subsequent analyses. To validate the RNA-seq results, qRT-PCR was conducted on selected target genes.

### 2.12. Western Blot Analysis

The uterine tissues were minced and homogenized in Lysis buffer (Solarbio, Beijing, China). The supernatants were collected after centrifugation of the homogenates. Protein concentrations were measured using a BCA assay kit (Solarbio, Beijing, China). Proteins (10 μg per lane) were separated by 10% sodium dodecyl sulfate-polyacrylamide gel electrophoresis and transferred to polyvinylidene fluoride membranes (Merck Millipore Ltd., Cork, Ireland) using a semi-dry transfer system (Biorad, Hercules, CA, USA). The membranes were blocked with 5% skimmed milk for 1.5 h, followed by incubation with primary antibodies and secondary antibodies (Table 2). Immunoreactive bands were visualized with a chemiluminescent imaging instrument (LI-COR, Linden, NJ, USA), and band intensities were quantified using ImageJ software 1.8.0 (NIH, Bethesda, MD, USA).

### 2.13. Statistical Analysis

Statistical analyses were performed using the Statistical Package for the Social Sciences 21.0 software (IBM, Armonk, NY, USA). Data were expressed as mean ± standard errors of the mean (SEM). Differences between groups were analyzed by one-way analysis of variance (ANOVA) followed by Tukey’s post hoc test. *p* < 0.05 represented statistical significance.

## 3. Results

### 3.1. Taurine Alleviated Pathological Injury and Inflammation in LPS-Induced Endometritis in Mice Through Decreasing MPO Activity and Maintaining Tight Junction Integrity

**Figure 1:**
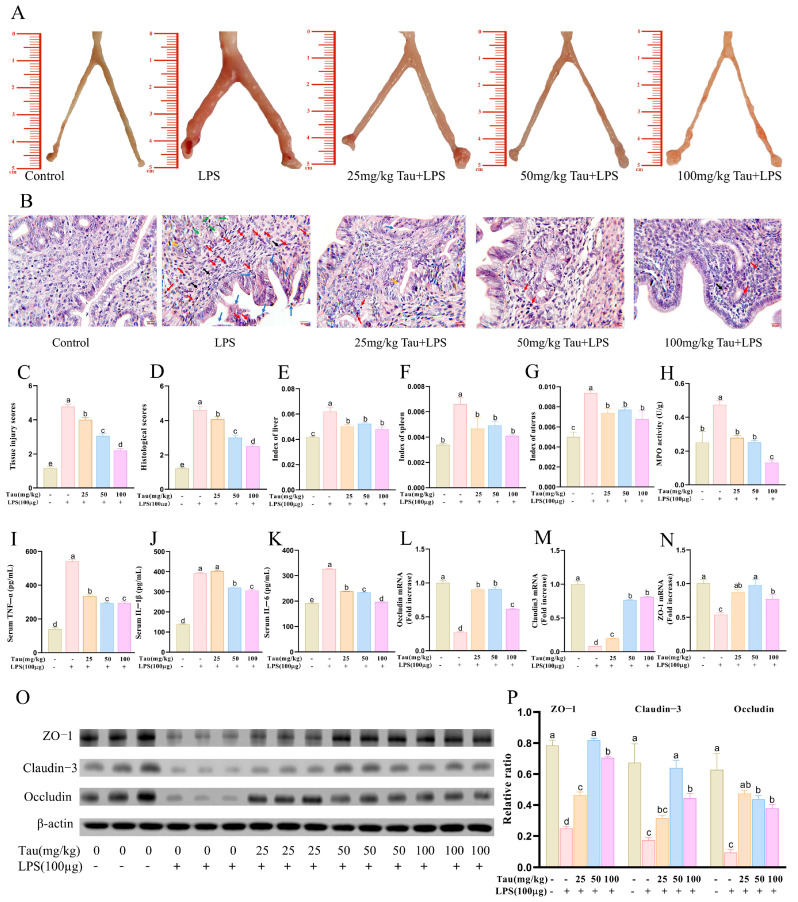
Effects of taurine on pathological injury and inflammation in LPS-induced endometritis in mice. (**A**) Morphological observation of pathological injury of uterine tissue in mice with endometritis. (**B**) Pathological injury of the uterine tissue in mice with endometritis was observed with HE staining. Red arrows represented neutrophil infiltration. Purple arrows represented lymphocyte infiltration. Blue arrows represented the shedding of endometrial epithelial cells. Black arrows represented capillary congestion and hemorrhage. Green arrows represented eosinophilic secretory deposits within uterine glands. Orange arrows represented the collapse of the uterine glands. (**C**,**D**) Tissue injury scores and histological scores. (**E**–**G**) The indices of the organs uterus, spleen, and liver in LPS-induced endometritis in mice. (**H**) MPO activities. (**I**–**K**) Serum levels of inflammatory cytokines TNF-α, IL-1β, and IL-6 in mice. (**L**–**N**) Tight junction mRNA expressions of ZO-1, Claudin-3, and Occludin were evaluated with qRT–PCR. (**O**,**P**) Illustrative bands and grayscale analysis results of tight junctions ZO-1, Claudin-3, and Occludin. Values were represented as mean ± SEM. No identical letter on top of the bar showed a remarkable difference (*p* < 0.05).

As shown in Figure 1A,C, the uterus of mice exhibited a pale yellow or light red coloration with a smooth surface, and the bilateral uterine horns appeared slender and symmetrical in the control group. The LPS group exhibited significant swelling, congestion, hemorrhage, rough surface, and asymmetrical morphology. In contrast, these pathological damages in the mice uteruses were ameliorated, and histological scores decreased (*p* < 0.05) with administration of taurine. HE staining was used to evaluate pathological injury (Figure 1B,D). It was demonstrated that uterine tissues in the LPS group exhibited severe inflammatory pathological damage, characterized by congestion in the submucosal layer and robust infiltration of neutrophils and macrophages. Epithelial detachment, fragmentation, and disorganized endothelial cell arrangement were observed in the endometrium. As well as the collapse and eosinophilic secretory deposits within the uterine glands. Notably, these pathological alterations were attenuated with taurine in a dose-dependent manner. As shown in Figure 1E–H, uterine index, spleen index, liver index, and MPO activities in mice uterine tissues were elevated (*p* < 0.05) with LPS treatment compared to the control group. Taurine treatment inhibited these changes (*p* < 0.05). Serum levels of inflammatory cytokines were detected (Figure 1I–K). LPS increased serum levels of TNF-α, IL-1β, and IL-6 compared to controls. Taurine treatment recovered TNF-α, IL-1β, and IL-6 serum levels in a dose-dependent manner. Then, tight junction-related protein expressions were evaluated (Figure 1L–P). Compared to controls, mRNA and protein expressions of ZO-1, Claudin-3, and Occludin decreased (*p* < 0.05) with LPS treatment, while taurine reversed the phenomenon (*p* < 0.05).

### 3.2. Taurine Alleviated Oxidative Stress in LPS-Induced Endometritis in Mice Through Improving Antioxidant Capacities

**Figure 2:**
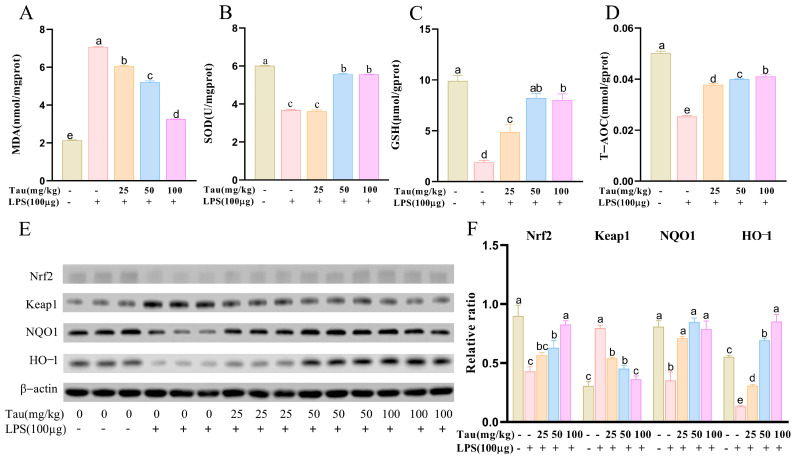
Effects of taurine on the oxidative stress in LPS-induced endometritis in mice. (**A**–**D**) The levels of MDA, SOD, GSH, and T-AOC. (**E**,**F**) Illustrative bands and grayscale analysis results of Nrf2, Keap1, NQO1, and HO-1. Values were represented as mean ± SEM. No identical letter on top of the bar showed a remarkable difference (*p* < 0.05).

Oxidative-stress-related factors were examined (Figure 2A–D). Compared to the control group, MDA contents were elevated (*p* < 0.05) following LPS stimulation. In comparison with the LPS group, MDA contents were reduced (*p* < 0.05) following 25, 50, and 100 mg/kg of taurine therapy. Concerning antioxidant factors, SOD, GSH, and T-AOC vitalities decreased (*p* < 0.05) with LPS administration compared to controls. SOD, GSH, and T-AOC levels were significantly increased (*p* < 0.05) after taurine treatment in a dose-dependent manner. Then, oxidative-stress-related proteins were examined (Figure 2E,F). With LPS stimulation, protein expressions of Nuclear factor erythroid 2-related factor 2 (Nrf2), NAD(P)H quinone dehydrogenase 1 (NQO1), and Heme oxygenase-1 (HO-1) decreased (*p* < 0.05), and Kelch-like ECH-associated protein 1 (Keap1) protein expression increased (*p* < 0.05) compared to the controls. Fortunately, taurine administration alleviated these changes.

### 3.3. Taurine Attenuated LPS-Induced Apoptosis in LPS-Induced Endometritis in Mice

**Figure 3:**
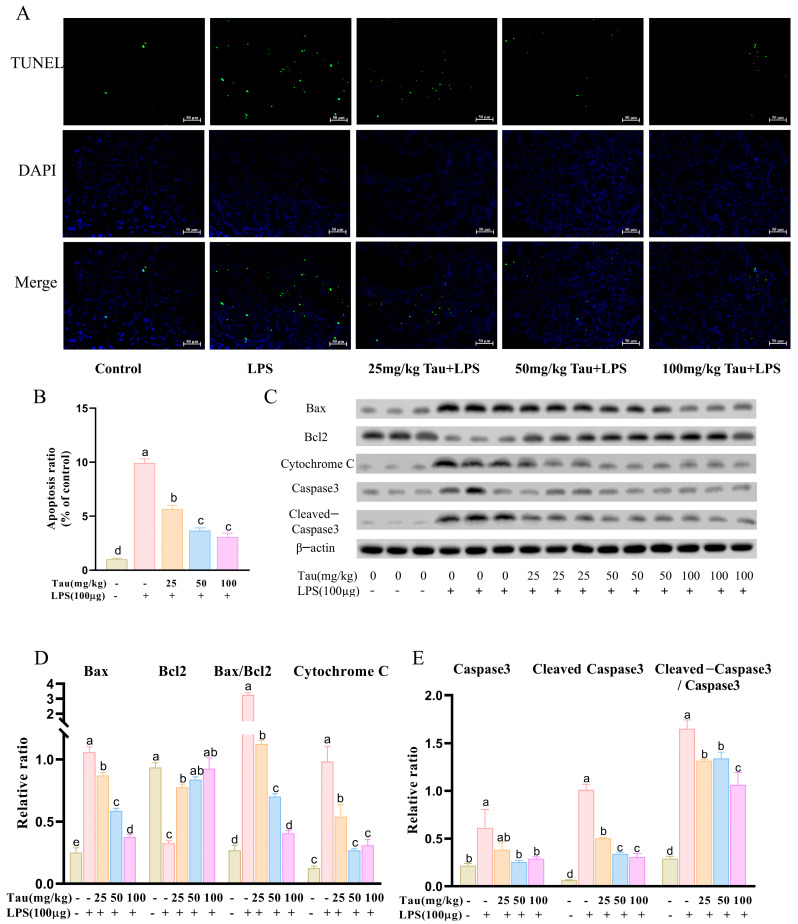
Effects of taurine on apoptosis in LPS-induced endometritis in mice. (**A**) The levels of apoptosis in uterine tissues across different groups of mice were assessed with terminal deoxynucleotidyl transferase-mediated dUTP nick-end labeling (TUNEL) staining. The green fluorescence indicated apoptotic cells, and the blue fluorescence represented the nuclei of all cells. (**B**) Quantitative analysis of the apoptosis ratio was conducted. (**C**–**E**) Illustrative bands and grayscale analysis results of Bax, Bcl-2, Cytochrome C, Caspase-3, and Cleaved-Caspase3. Values were represented as mean ± SEM. No identical letter on top of the bar showed a remarkable difference (*p* < 0.05).

The levels of apoptosis in uterine tissues were assessed with TUNEL staining. The results were shown in Figure 3A,B. With stimulation of LPS, the levels of apoptosis in uterine tissues were upregulated (*p* < 0.05) compared to the control group. In contrast, apoptosis levels of the uterus tissues decreased (*p* < 0.05) by 25, 50, and 100 mg/kg of taurine treatment. As shown in Figure 3C–E, apoptosis-related protein expressions were examined. Compared to the control group, expressions of B-cell lymphoma-2-associated x protein (Bax), Cytochrome C, cysteine-aspartic acid protease-3 (Caspase-3), and cleaved-Caspase3 proteins increased (*p* < 0.05), and B-cell lymphoma gene-2 (Bcl-2) protein expression reduced (*p* < 0.05) by treatment with LPS. Bax/Bcl2 and Cleaved-Caspase3/Caspase3 ratios were also elevated (*p* < 0.05). However, different doses of taurine reversed changes in Bax, Cytochrome C, Cleaved-Caspase3, and Bcl2, as well as Bax/Bcl2 and Cleaved-Caspase3/Caspase3 ratios (*p* < 0.05). In addition, Caspase3 expression decreased (*p* < 0.05) with 50 and 100 mg/kg of taurine treatment.

### 3.4. Taurine Regulated Uterus Microbiota Composition in LPS-Induced Mice Endometritis

**Figure 4:**
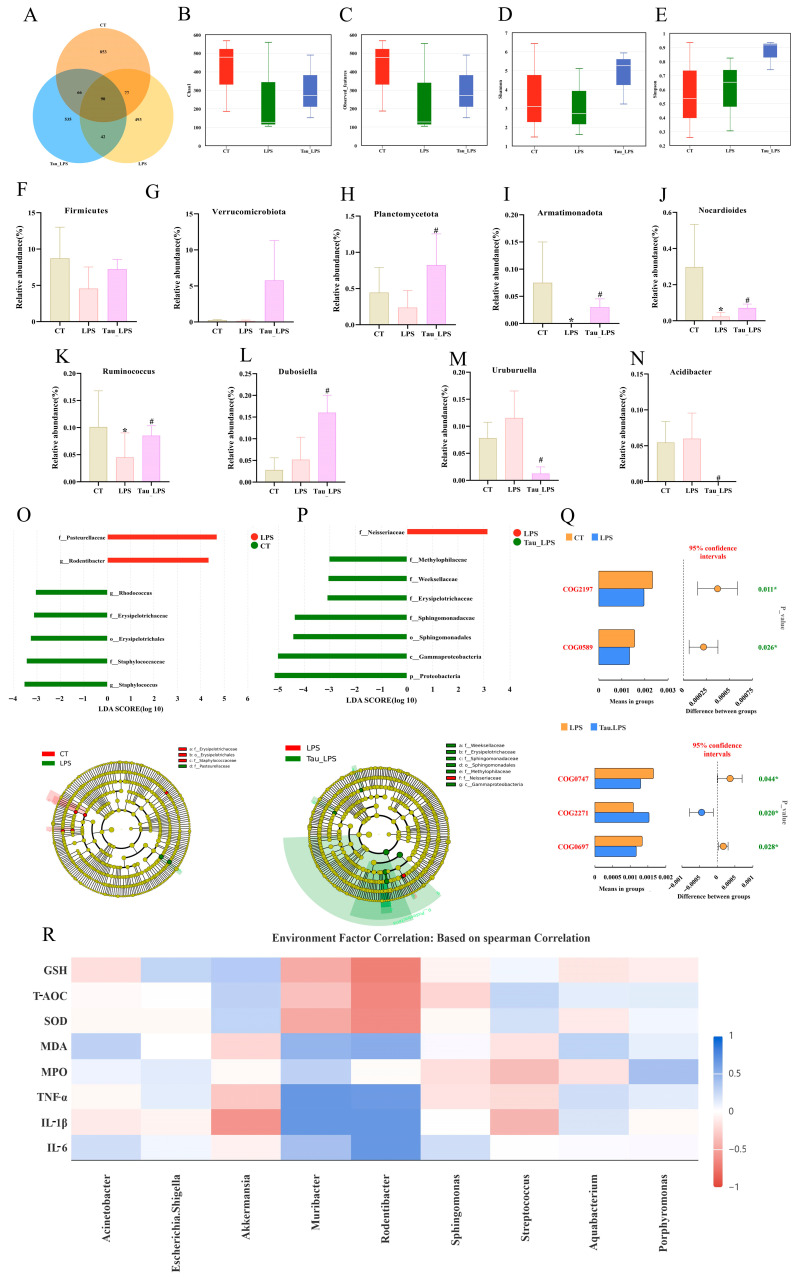
Effect of taurine on uterine microbiota in mice. (**A**) The Venn plot for operational taxonomic units (OTUs) among the CT, LPS, and Tau_LPS groups. (**B**–**E**) The microbial community richness and alpha diversity indices. (**F**–**I**) Alterations in the relative abundance of *Proteobacteria*, *Verrucomicrobiota*, *Planctomycetota*, and *Armatimonadota* at the phylum level across groups. (**J**–**N**) Alterations in the relative abundance of *Nocardioides*, *Ruminococcus*, *Dubosiella*, *Uruburuella*, and *Acidibacter* at the genus level across groups. (**O**,**P**) LEfSe analysis (LDA score > 3). (**Q**) T-test of PICRUSt2 analysis for functional prediction. (**R**) The correlation analysis with Spearman between gut microbiota and inflammation and oxidative stress markers in LPS-induced endometritis in mice. Values were represented as mean ± SEM. * *p* < 0.05 compared to the CT group; # *p* < 0.05 compared to the LPS group. CT: control group; LPS: LPS group; Tau_LPS: 100 mg/kg taurine + LPS group.

An analysis using a Venn diagram found that there were 853, 493, and 535 unique operational taxonomic units in the control (CT) group, the LPS group, and the 100 mg/kg of taurine treatment (Tau_LPS) group, respectively (Figure 4A). Microbial alpha diversity was assessed using the Chao1, observed features, Shannon, and Simpson indices (Figure 4B–E). Compared to the control group, both Chao1 and observed features indices reduced with LPS administration, indicating diminished microbial richness. These indices were restored with taurine intervention, albeit without statistical significance. In terms of diversity, the Shannon index decreased with LPS treatment, whereas both the Shannon and Simpson indices increased with the administration of taurine. MetagenomeSeq analysis was performed to evaluate taxonomic shifts at the phylum and genus levels (Figure 4F–N). The relative abundances of Firmicutes, Verrucomicrobiota, and Planctomycetota at the phylum level exhibited a declining trend by treating LPS. In addition, the relative abundance of Armatimonadota was reduced (*p* < 0.05). With taurine intervention, the relative abundances of Firmicutes and Verrucomicrobiota showed a non-significant increase, whereas the relative abundances of Planctomycetota and Armatimonadota were significantly restored (*p* < 0.05). At the genus level, the relative abundances of Nocardioides and Ruminococcus decreased (*p* < 0.05), and the relative abundances of Dubosiella, Uruburuella, and Acidibacter increased with LPS exposure. The relative abundances of Nocardioides, Ruminococcus, and Dubosiella were elevated (*p* < 0.05), and the relative abundances of Uruburuella and Acidibacter were reduced (*p* < 0.05) with the intervention of taurine. To further clarify the differential characteristics of uterine microbial communities among mice in various groups, linear discriminant analysis effect size (LEfSe) was employed for in-depth investigation (Figure 4O,P). The results revealed that significant differences existed in the uterine microbial communities at all taxonomic levels among the CT, LPS, and Tau_LPS groups. Specifically, the relative abundances of Pasteurellaceae and Rodentibacter in the uterine tissue of mice increased after LPS treatment. In contrast, taurine intervention reshaped the structure of dominant microbial communities in the uterus, as evidenced by the enriched taxa including Methylophilaceae, Weeksellaceae, Erysipelotrichaceae, Sphingomonadaceae, Sphingomonadales, Gammaproteobacteria, and Proteobacteria. As shown in Figure 4Q, based on the T-test of PICRUSt2 analysis for functional prediction, compared with the control group, the expression levels of DNA-binding response regulator and Nucleotide-binding universal stress protein in mouse uterine tissue decreased after LPS treatment. In contrast, after taurine intervention, the activities of the ABC-type transport system and Permease of the drug/metabolite transporter (DMT) superfamily in mouse uterine tissue decreased, while the activity of Sugar phosphate permease increased. Furthermore, Spearman correlation analysis was performed to systematically explore the association between inflammatory factors, oxidative stress indicators, and core uterine microbial taxa. As shown in Figure 4R, the relative abundances of Akkermansia and Streptococcus were positively correlated with the antioxidant indicators (GSH, T-AOC, SOD), and negatively correlated with the pro-inflammatory cytokines (IL-1β, TNF-α) and oxidative stress markers (MDA). On the contrary, the abundances of Muribacter and Rodentibacter exhibited positive correlations with IL-6, IL-1β, TNF-α, and MDA, but negative correlations with GSH, T-AOC, and SOD.

### 3.5. The Potential Mechanism of Taurine in LPS-Induced Mice Endometritis Was Analyzed by RNA Sequencing

**Figure 5:**
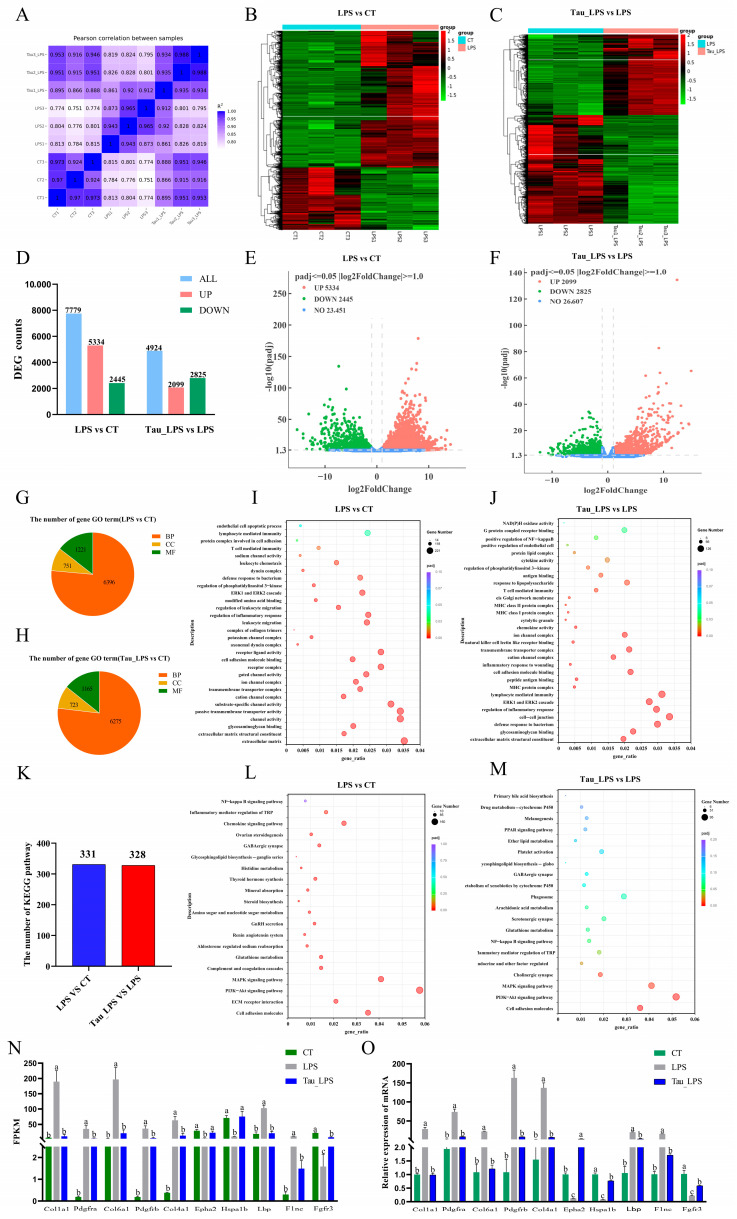
The potential mechanism of taurine in LPS-induced endometritis in mice was analyzed by RNA-seq. (**A**) Heat map of correlation between groups. (**B**,**C**) Clustering heatmap of DEGs in differential comparison groups. (**D**) Counts of DEGs in differential comparison groups. (**E**,**F**) Volcano plots of DEGs. (**G**–**J**) GO enrichment analysis of the DEGs quantity distribution chart and bubble chart. (**K**–**M**) Statistical chart and bubble chart of the KEGG pathway number of DEGs in the ovaries of mice in each group. (**N**,**O**) The FPKM values of part DEGs in each group were verified by qRT-PCR. Values were represented as mean ± SEM. No identical letter on top of the bar showed a remarkable difference (*p* < 0.05). CT: control group; LPS: LPS group; Tau_LPS: 100 mg/kg taurine + LPS group.

To investigate the potential mechanism of taurine in alleviating LPS-induced endometritis in mice, the uterine tissues from the control (CT) group, the LPS group, and the 100 mg/kg of taurine treatment (Tau_LPS) group were used for RNA-sequencing research. The correlation coefficients between CT, LPS, and Tau_LPS groups were greater than 0.87, showing the sequencing results were reliable (Figure 5A). The gene cluster heat map showed that the gene expressions significantly changed in different groups (Figure 5B,C). Compared to those in the control group, a total of 7779 differentially expressed genes (DEGs) were exhibited in the LPS group, including 5334 upregulated and 2445 downregulated. In comparison with the LPS group, the Tau_LPS group showed 4924 DEGs, with 2099 upregulated and 2825 downregulated (Figure 5D–F). The identified DEGs were analyzed based on the GO analysis database. Compared to the control group, the LPS group showed enrichment in 8368 terms, including 6396 biological process (BP) terms, 751 cellular component (CC) terms, and 1221 molecular function (MF) terms. Compared to the LPS group, 8163 terms were enriched in the Tau_LPS group, consisting of 6275 BP terms, 723 CC terms, and 1165 MF terms. Based on the GO enrichment results, it is suggested that LPS might induce uterine tissue damage in mice through pathways mainly associated with immunity, including leukocyte migration, inflammatory response, ERK1 and ERK2 cascade, regulation of phosphatidylinositol 3-kinase signaling, defense response to bacteria, T cell-mediated immunity, lymphocyte-mediated immunity, and endothelial cell apoptotic process. Taurine appeared to mitigate LPS-induced tissue injury by modulating these pathways (Figure 5G–J). KEGG pathway enrichment analysis of DEGs demonstrated that 331 significantly enriched pathways were screened out in the LPS group compared to controls. In comparison with the LPS group, 328 enriched pathways were screened out in the Tau_LPS group. Among the top 20 endometritis-related pathways, it was shown that the pathways by which taurine alleviated LPS damage were mainly related to cell adhesion molecules, the PI3K-Akt signaling pathway, the MAPK signaling pathway, inflammatory mediator regulation of TRP channels, the NF-κB signaling pathway, and Glutathione metabolism (Figur 5K–M). Furthermore, ten key genes (Col1a1, Pdgfra, Col6a1, Pdgfrb, Col4a1, Epha2, Hspa1b, Lbp, Flnc, and Fgfr3) from the PI3K-AKT/MAPK/NF-κB signaling pathway were selected for qRT-PCR validation. The results demonstrated that mRNA expressions of these genes were highly consistent with the fragments per kilobase of transcript per million mapped reads (FPKM) value trends obtained from RNA sequencing (Figure 5N,O), further confirming the reliability of the transcriptome sequencing results.

### 3.6. Taurine Alleviated LPS-Induced Endometritis in Mice by Inhibiting PI3K-Akt/MAPK/NF-κB Signaling Pathways

**Figure 6:**
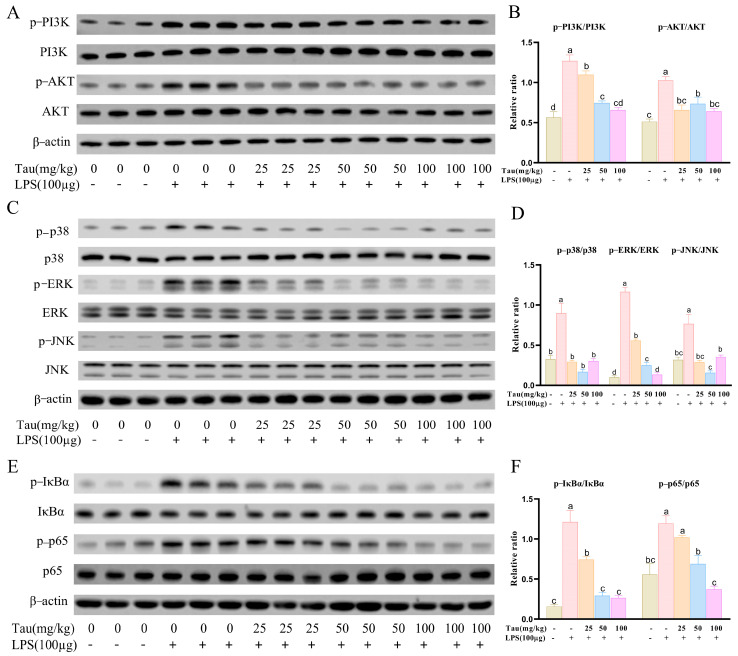
Effects of taurine on PI3K-AKT/MAPK/NF-κB pathways in LPS-induced endometritis in mice. (**A**,**B**) Illustrative bands and grayscale analysis results of p-PI3K, PI3K, p-Akt, and Akt in the PI3K-AKT pathway. (**C**,**D**) Illustrative bands and grayscale analysis results of p-p38, p38, p-ERK, ERK, p-JNK, and JNK in the MAPK pathway. (**E**,**F**) Illustrative bands and grayscale analysis results of p-IκBα, IκBα, p-p65, and p65 in the NF-κB pathway. Values were represented as mean ± SEM. No identical letter on top of the bar showed a remarked difference (*p* < 0.05).

To further validate the roles of PI3K-Akt/MAPK/NF-κB signaling pathway in taurine modulating mice endometritis, related protein expressions were determined. It was shown that p-PI3K and p-Akt protein levels increased (*p* < 0.05) in the uterus tissues with LPS treatment, and decreased (*p* < 0.05) by taurine intervention (Figure 6A,B). Similarly, phosphorylation levels of p38 mitogen-activated protein kinase (p38), extracellular signal-regulated kinase (ERK), and c-Jun N-terminal kinase (JNK) in the MAPK pathway and phosphorylation levels of inhibitor of kappa B alpha (IκBα) and nuclear factor NF-κB p65 subunit (p65) in the NF-κB pathway were enhanced (*p* < 0.05) with LPS treatment compared to the control group. However, the phosphorylation status of p38, ERK, JNK, p65, and IκBα were inhibited (*p* < 0.05) by 25, 50, and 100 mg/kg of taurine treatment (Figure 6C–F).

## 4. Discussion

Escherichia coli, a Gram-negative commensal bacterium in the intestinal tract, is pathogenic through virulence factors such as LPS [15]. In dairy cattle, pathogenic Escherichia coli is the primary causative agent of endometritis, of which the LPS component activates the TLR4/NF-κB signaling pathway, triggering endometrial inflammation [16]. This inflammatory response severely compromises reproductive function, leading to delayed postpartum estrus, increased conception failures, and imposing economic burdens [17]. Current therapeutic strategies for endometritis predominantly rely on antibiotics; however, prolonged use of antibiotics not only fosters antimicrobial resistance but also poses potential risks to both bovine and human health [18]. Consequently, exploring efficacious and safe antibiotic alternatives has emerged as a widespread issue that needs attention and solutions.

Taurine, a sulfur-containing amino acid, exhibits remarkable antioxidant, anti-inflammatory, and immunomodulatory properties, which mitigate damage and promote repair of tissue [19]. So far, taurine has been widely used to prevent and treat animal diseases [20]. However, reports about taurine in the treatment of endometritis are limited. Thus, the research is conducted. Mice are standard model animals. Jiang et al. [21] and Ma et al. [22] have reported that the research results of the mouse endometritis model are consistent with those of the dairy cow endometritis model. This conclusion indicates that mice can be used for research related to dairy cow endometritis; therefore, in this study, a mouse endometritis model is established, and subsequent experiments are carried out based on this model. To investigate the effects of taurine on endometritis in dairy cows, mouse endometritis models were established with LPS treatment, and 25, 50, and 100 mg/kg of taurine were administered. It is confirmed that taurine can alleviate LPS-induced endometritis in mice through ameliorating inflammatory injury, oxidative stress, and apoptosis and recovering the uterus microbiota composition. The underlying mechanism is mainly mediated by the PI3K-Akt/MAPK/NF-κB pathways.

Pathological manifestations can reflect the extent of tissue damage [23,24]. Consistent with the study of Wang et al. [25], marked swelling, hyperemia, and hemorrhage of the uterine tissues and elevated organ weight indices are observed with LPS treatment. In agreement with the survey of Nazmy et al. [26] and Surai et al. [27], pathological changes were mitigated with taurine treatment. As neutrophil activation markers and core pro-inflammatory factors [28,29,30], MPO activities of mice uterine tissues and serum levels of IL-1β, IL-6, and TNF-α elevate with LPS treatment, indicating an inflammatory response, which is also confirmed with histopathological changes in uterine tissue sections. In this study, MPO activities and pro-inflammatory factor levels decreased with taurine administration, demonstrating the efficacy of taurine in alleviating neutrophil infiltration and tissue inflammatory injury. As a robust barrier, tight junctions can be impaired under pathological conditions [31,32]. The downregulation of ZO-1, claudin-3, and occludin within the tight junction complex was confirmed, while taurine reversed the phenomenon. These findings are consistent with the studies of Li et al. [33] and Li et al. [34]. Therefore, it is confirmed that taurine alleviates LPS-induced inflammation of mouse uterine tissue by relieving pathological injury, decreasing MPO and pro-inflammatory cytokine levels, and maintaining the integrity of tight junctions.

With the activation of inflammation, it is also accompanied by oxidative stress and apoptosis induced by ROS accumulation and mitochondrial dysfunction [35,36,37]. Elevated MDA contents coupled with reduced SOD and GSH activities are recognized as hallmarks of oxidative stress [38,39]. In this study, MDA contents increased, and SOD, GSH, and T-AOC levels decreased with LPS treatment, leading to abnormal expression of Nrf2, Keap1, HO-1, and NQO1. As a master transcriptional regulator of antioxidant responses, Nrf2 maintains redox equilibrium and protects cells from oxidative damage by activating antioxidant gene expression [40]. Additionally, apoptosis-related proteins Bax and Bcl2 were abnormally expressed, Cytochrome C was released, and caspase cascade reactions were activated with LPS stimulation in the study, showing the occurrence of apoptosis in LPS-induced mice uterine tissues. It was also confirmed by TUNEL analysis. Fortunately, taurine administration alleviated these changes, demonstrating its protective role against LPS-induced mice uterine oxidative stress and apoptosis through restoring levels of oxidative-stress-related proteins and antioxidants and restoring apoptosis-related protein levels and blocking caspase cascade reactions, which are in line with reports of Piao et al. [41], Ghanim et al. [42], and Wu et al. [43].

As a vital component of uterine tissue, the microbiota plays crucial roles in immune homeostasis, inflammatory responses, and endocrine regulation for reproductive health and pregnancy outcomes [44]. Bacterial dysbiosis is a primary characteristic of endometritis [45]. The microbial species richness, diversity, and abundances of the Firmicutes and Bacteroidetes are reduced in animals with endometritis [46,47]. Consistent with these reports, it was demonstrated that Chao1, observed features, and Shannon indices, as well as Firmicutes and Bacteroidetes abundances, decreased in mice with endometritis in this study. Microbiota changes at the genus level were also investigated. The abundances of Nocardioides and Ruminococcus were reduced, and Uruburuella and Acidibacter increased with LPS treatment. Taurine intervention reversed these microbial alterations and additionally increased the abundance of Dubosiella. Previous studies indicate that Nocardioides, Ruminococcus, and Dubosiella produce various bioactive natural metabolites with anti-inflammatory, metabolic regulatory, growth-promoting, and antimicrobial properties against pathogens [48,49,50]. Conversely, Uruburuella acts as an opportunistic pathogen implicated in malignant biliary obstruction [51] and porcine pneumonia and pericarditis [52]. Acidibacter is primarily associated with heavy metal degradation and soil-transmitted diseases like schistosomiasis [53]. A novel finding was presented that an association between altered Acidibacter abundance and endometritis was revealed in this study. According to established literature, Rodentibacter has been classified under the family Pasteurellaceae [54,55]. It has been reported to act as an opportunist or even a primary pathogen in immunocompromised hosts under conditions such as stress, viral infection, or adverse environments [56]. Muribacter, originally isolated from the oral cavity of mice [57], is examined in this study. Our findings indicated that the abundances of Muribacter and Rodentibacter were positively correlated with levels of pro-inflammatory cytokines, MPO, and MDA, while showing a negative correlation with antioxidative stress markers. Therefore, the relative abundances of Muribacter and Rodentibacter might serve as potential biomarkers for endometritis in mice. Akkermansia has been shown to alleviate metabolic syndrome and intestinal mucosal damage by inducing anti-inflammatory responses and modulating gut homeostasis [58]. Its potential as an immunomodulatory probiotic for autoimmune and chronic inflammatory diseases has been explored in experimental models [59]. Moreover, certain species within the genus Streptococcus have demonstrated beneficial probiotic properties and are widely applied in the prevention and treatment of oral diseases [60,61]. Consistent with these reports, the present study revealed that Akkermansia and Streptococcus exhibited notable probiotic effects, suggesting that taurine might ameliorate LPS-induced endometritis in mice by modulating the abundance of these bacterial genera in the uterine microbiota. In addition, the functional prediction results further suggest that taurine might exert its protective effects by decreasing the activities of the ABC-type transport system and permease of the drug/metabolite transporter (DMT) superfamily and increasing the activity of sugar phosphate permease.

To investigate the mechanism underlying taurine-mediated alleviation of LPS-induced endometritis in mice, transcriptome sequencing is performed. 7779 DEGs were revealed in the LPS group by transcriptomic profiling, while 4924 DEGs were identified in the taurine group. It is demonstrated that the protective effects of taurine primarily target immunoregulatory processes and the PI3K-Akt/MAPK/NF-κB signaling axis by GO enrichment and KEGG pathway analyses. The PI3K/AKT pathway regulates inflammatory responses by recruiting and activating innate immune cells and is associated with the pathogenesis of diseases [62]. Among pathway-associated genes, Col1a1, Pdgfra, Col6a1, Pdgfrb, Col4a1, Lbp, Flnc, and Fgfr3 were upregulated with pathway activation signatures. The collagen α1-chain genes (Col1a1, Col6a1, and Col4a1) are observed to establish a signaling platform through interactions with cell surface receptors [63]. Receptor tyrosine kinases (Pdgfra, Pdgfrb, Fgfr3) undergo dimerization upon ligand binding, resulting in phosphorylation of intracellular tyrosine residues. This phosphorylation event triggered the PI3K pathway, generating phosphatidylinositol (3,4,5)-trisphosphate, which initiates cellular proliferation and survival programs [64]. PI3K/Akt pathway activation is further shown to propagate signaling through multiple cascades. Downstream RAF kinase stimulation activates the MAPK pathway [65]. Expression levels of anti-apoptotic proteins Bcl-xL and Bcl-2 are modulated [66]. Phosphorylation of IκB kinase (IKK) liberates NF-κB from inhibition, enabling its nuclear translocation and activation of pro-inflammatory gene transcription. Under exacerbated inflammation, lipopolysaccharide-binding protein (LBP) activates the MyD88-dependent NF-κB pathway. Then, pro-inflammatory cytokines are released [67]. Filamin C (Flnc) potentiated sustained PI3K-Akt activation by anchoring integrins to focal adhesion kinase (FAK) and amplified inflammatory signaling through p38/ERK activation in the MAPK pathway [68]. NADPH oxidase hyperactivation and mitochondrial dysfunction are associated with progressive inflammatory cascades, leading to ROS generation and oxidative stress damage [35,36]. Nam et al. [69] and Jiang et al. [70] also demonstrate that LPS induces tissue damage by activating the PI3K-Akt/MAPK/NF-κB pathway. Taurine administration significantly suppresses the aberrant expression of these genes, attenuating inflammatory responses, oxidative stress, and apoptosis. The results are consistent with those reports of Shi et al. [71] and Naderi et al. [72]. Collectively, it is indicated that taurine alleviates LPS-induced endometritis through inhibition of the PI3K-Akt/MAPK/NF-κB pathway. The study provides valuable insights and practical references for therapeutic strategies against endometritis. However, the investigation is confined to in vivo experimental models due to time constraints. Future studies should be extended to in vitro systems combined with pathway-specific inhibitors for a more comprehensive understanding.

## 5. Conclusions

Above all, taurine alleviates inflammatory injury, oxidative stress, apoptosis and uterus microbiota dysregulation in mice endometritis through modulating the PI3K-AKT/MAPK/NF-κB pathways. It is confirmed that taurine has a therapeutic effect for endometritis and the possible mechanism in this research. Hence, taurine could be a candidate drug for bovine endometritis.

## Figures and Tables

**Table 1:** Primers used in quantitative real-time PCR analysis.

Genes		Primer Sequence (5′–3′)	Product Sizes (bp)	Gene Bank Accession No.
*Col1a1*	F	AGCACGTCTGGTTTGGAGAG	112	NM_007742.4
	R	GACATTAGGCGCAGGAAGGT		
*Pdgfra*	F	GCCTGAGCTTTGAGCGACG	81	NM_001083316.2
	R	AGCTGAGGACCAGAAAGACCT		
*Col6a1*	F	AAAGGCACCTACACCGACTG	135	NM_009933.5
	R	GCATGGTTCCTTGTAGCCCT		
*Pdgfrb*	F	AGAAGCCACGCTATGAGATCC	100	NM_008809.2
	R	GAGTCGTAAGGCAACTGCAC		
*Col4a1*	F	AACAACGTCTGCAACTTCGC	136	NM_009931.2
	R	CTTCACAAACCGCACACCTG		
*Epha2*	F	ACATCATGGACGACATGCCT	147	NM_010139.3
	R	TACAGTCTCGCACCGTGAAC		
*Hspa1b*	F	CACCATCGAGGAGGTGGATTA	104	NM_010478.3
	R	TTGACAGTAATCGGTGCCCAA		
*Lbp*	F	GAGGCCTGTGTAAGTGAGCA	117	NM_8489.2
	R	AAATCACGGTCTCTCCTCGC		
*Flnc*	F	TGTGGCAGAAGCCTGTAACC	97	NM_001081185.2
	R	CACCTTGAAGTCAGCCACCT		
*Fgfr3*	F	GTGCGATCCACTCCGGC	115	NM_001163215.2
	R	GCTAGAGCCCAACTCACCAC		
*Occludin*	F	TCTTTCCTTAGGCGACAGCG	88	NM_001360536.1
	R	AGATAAGCGAACCTGCCGAG		
*ZO-1*	F	AGACGCCCGAGGGTGTAG	146	NM_001163574.2
	R	TGGGACAAAAGTCCGGGAAG		
*Claudin-3*	F	GTAAACAGAGCCGGTTTCGG	140	NM_204202.2
	R	CACGCGTAACAGGGAGAGAA		
*GAPDH*	F	AGGTCGGTGTGAACGGATTTG	164	NM_001289726.1
	R	GGGGTCGTTGATGGCAACA		

**Table 2:** Antibodies used in Western blot analysis.

Antibodies	Manufacturers	Catalog Numbers	Dilution Ratios
β-actin	Bioss (Bioss Inc., Woburn, MA, USA)	bs-0061R	1:5000
p65	Bioss	bs-0465R	1:1000
phospho-p65	Bioss	bs-0982R	1:1000
IκBα	Bioss	bs-1287R	1:1000
phospho-IκBα	Bioss	Bsm-52169R	1:1000
p38	CST (Cell Signaling Technology, Inc., Danvers, MA, USA)	#8690	1:1000
phospho-p38	CST	#4a511	1:1000
ERK	CST	#4695T	1:1000
phospho-ERK	CST	#4370T	1:2000
JNK	CST	#9252T	1:1000
phospho-JNK	CST	#4668T	1:1000
HO-1	Bioss	bs-2075R	1:1000
NQO1	Bioss	bs-2184R	1:2000
Keap1	Bioss	bs-4900R	1:2000
Nrf2	Bioss	bs-1074R	1:500
Bcl-2	Bioss	bs-4563R	1:1000
Bax	Bioss	bs-0127R	1:1000
Caspase-3	Bioss	bs-0081R	1:1000
Cleaved-Caspase3	HUABIO (HUABIO LLC, Fremont, CA, USA)	ET1602-47	1:2000
Cytochrome C	Bioss	bsm-52050R	1:1000
PI3K	GenuIN (Gen-Insight Biotechnology Co., Ltd., Suzhou, China)	51138	1:1000
Phospho-PI3K	GenuIN	U1011	1:1000
AKT	Diagbio (Diagbio Technology Co., Ltd., Hangzhou, China)	db11977	1:1000
Phospho-AKT	Diagbio	db13991	1:1000
Goat Anti-Rabbit IgG H&L, HRP conjugated	Bioss	bs-40295G-HRP	1:5000

## Data Availability

All data in this article are presented in the article.

## Abbreviations

PI3K-AKT, Phosphatidylinositol 3-kinase-protein kinase B; LPS, lipopolysaccharide; NF-κB, nuclear factor kappa B; MAPK, mitogen-activated protein kinase; SOD, superoxide dismutase; GSH, glutathione; Caspase-3, Cysteine-aspartic acid protease-3; Tau, taurine; HE, hematoxylin and eosin; MPO, Myeloperoxidase; TNF-α, tumor necrosis factor alpha; IL-6, interleukin-6; IL-1β, interleukin-1β; DEGs, differentially expressed genes; Nrf2, nuclear factor erythroid 2-related factor 2; HO-1, heme oxygenase-1; Keap1, Kelch-like ECH-associated protein 1; NQO1, NAD(P)H quinone dehydrogenase 1; Bax, B-cell lymphoma-2 associated-x; Bcl-2, B-cell lymphoma gene-2; Caspase-3, cysteine-aspartic acid protease-3; BP, biological process; CC, cellular component; MF, molecular function; ERK, extracellular signal-regulated kinases; p38, p38 mitogen-activated protein kinases; JNK, c-Jun N-terminal kinase; IκBα, inhibitor of kappa B alpha; p65, nuclear factor kappa B p65 subunit.
